# Genome-wide association study of endo-parasite phenotypes using imputed whole-genome sequence data in dairy and beef cattle

**DOI:** 10.1186/s12711-019-0457-7

**Published:** 2019-04-18

**Authors:** Alan J. Twomey, Donagh P. Berry, Ross D. Evans, Michael L. Doherty, David A. Graham, Deirdre C. Purfield

**Affiliations:** 1 Animal and Grassland Research and Innovation Centre, Teagasc, Moorepark, Fermoy, Co. Cork Ireland; 2 School of Veterinary Medicine, University College Dublin, Belfield, Dublin 4, Ireland; 3 Irish Cattle Breeding Federation, Highfield House, Bandon, Co. Cork Ireland; 4 Animal Health Ireland, Carrick on Shannon, Co. Leitrim Ireland

## Abstract

**Background:**

Quantitative genetic studies suggest the existence of variation at the genome level that affects the ability of cattle to resist to parasitic diseases. The objective of the current study was to identify regions of the bovine genome that are associated with resistance to endo-parasites.

**Methods:**

Individual cattle records were available for *Fasciola hepatica*-damaged liver from 18 abattoirs. Deregressed estimated breeding values (EBV) for *F. hepatica*-damaged liver were generated for genotyped animals with a record for *F. hepatica*-damaged liver and for genotyped sires with a least one progeny record for *F. hepatica*-damaged liver; 3702 animals were available. In addition, individual cow records for antibody response to *F. hepatica* on 6388 genotyped dairy cows, antibody response to *Ostertagia ostertagi* on 8334 genotyped dairy cows and antibody response to *Neospora caninum* on 4597 genotyped dairy cows were adjusted for non-genetic effects. Genotypes were imputed to whole-sequence; after edits, 14,190,141 single nucleotide polymorphisms (SNPs) and 16,603,644 SNPs were available for cattle with deregressed EBV for *F. hepatica*-damaged liver and cows with an antibody response to a parasitic disease, respectively. Association analyses were undertaken using linear regression on one SNP at a time, in which a genomic relationship matrix accounted for the relationships between animals.

**Results:**

Genomic regions for *F. hepatica*-damaged liver were located on *Bos taurus* autosomes (BTA) 1, 8, 11, 16, 17 and 18; each region included at least one SNP with a p value lower than 10^−6^. Five SNPs were identified as significant (q value < 0.05) for antibody response to *N. caninum* and were located on BTA21 or 25. For antibody response to *F. hepatica* and *O. ostertagi*, six and nine quantitative trait loci (QTL) regions that included at least one SNP with a p value lower than 10^−6^ were identified, respectively. Gene set enrichment analysis revealed a significant association between functional annotations related to the olfactory system and QTL that were suggestively associated with endo-parasite phenotypes.

**Conclusions:**

A number of novel genomic regions were suggestively associated with endo-parasite phenotypes across the bovine genome and two genomic regions on BTA21 and 25 were associated with antibody response to *N. caninum*.

**Electronic supplementary material:**

The online version of this article (10.1186/s12711-019-0457-7) contains supplementary material, which is available to authorized users.

## Background

Parasitic diseases are highly prevalent worldwide in cattle production systems [1] and this prevalence is forecasted to increase further as a repercussion of the anticipated change in climate [2]. In Ireland, between 75.4% [3] and 83% [4] of the Irish dairy herds, show evidence of exposure to *Fasciola hepatica*. In addition, parasitic diseases are associated with significant economic losses in both dairy and beef cattle [5–7]. Details on endo-parasite diseases in cattle are in Sekiya et al. [1]. Using pedigree-based relationships between individuals, Twomey et al. [8] documented the presence of genetic variation for two phenotypes due to *F. hepatica* in cattle and proposed that selection and breeding animals resistant to *F. hepatica* would complement current control strategies for *F. hepatica* (i.e., anthelmintic treatments; [9]). Genetic variability has also been documented for other endo-parasites in cattle such as *Ostertagia ostertagi* [10, 11] and *Neospora caninum* [10, 12]. Since genetic gain is a function of selection accuracy [13], the accuracy of traditional genetic evaluations that exploit only pedigree relationships are hindered by the lack of available phenotypic data; this is especially true for low heritability traits. Although the study of Twomey et al. [8] was limited by the low accuracy of the genetic evaluations, they reported a 6% unit difference in the prevalence of *F. hepatica*-damaged liver between cows with an estimated breeding value (EBV) for *F. hepatica* in the top 10% versus the bottom 10%; the mean EBV reliability of those animals was 0.18. It is hypothesised that the inclusion of genomic information within these evaluations will improve the reliability of the EBV for *F. hepatica* and thus improve the prediction of whether or not an animal will have *F. hepatica*-damaged liver.

Few studies have attempted to locate genomic regions associated with endo-parasitic diseases in cattle. Using a genotyping panel of only 153 microsatellite markers, Coppieters et al. [11] documented that *Bos taurus* autosomes (BTA) 9 and 19 harboured quantitative trait loci (QTL) associated with nematode burden in 768 Dutch dairy cows that were selected to be in the top 10% and bottom 10% of their respective sire family (n = 12) for faecal egg count (FEC). Using 305 Aberdeen Angus calves genotyped for 190 microsatellite markers only, Kim et al. [14] reported that regions on BTA8 and 12 were associated with FEC. To the best of our knowledge, Kim et al. [15] is the only available study that used dense genome-wide SNP genotypes (31,165 SNPs after edits) to map genomic regions associated with endo-parasitic diseases in cattle; they reported that 12 regions in the bovine genome contributed to significant genetic variation in FEC.

The objective of our study was to identify single nucleotide polymorphisms (SNPs) associated with three endo-parasitic diseases in cattle using imputed whole-genome sequence data in a large multi-breed population. Results from this study will improve our understanding of the underlying genetic mechanisms of resistance in cattle to parasitic diseases. This could contribute to enhance the development of further control strategies for parasitic diseases, such as vaccine development, and to improve diagnostics of parasitic diseases. In addition, our results provide information that would be useful in future studies that attempt to increase the accuracy of genetic evaluations, through the exploration of targeted genomic regions.

## Methods

The phenotypic data analysed in this study originated from two sources: (1) national abattoir cattle data containing records for *F. hepatica*-damaged liver, and (2) serological testing of individual cows in 68 dairy herds for endo-parasites.

### Phenotypic data for *F. hepatica*-damaged liver

Individual animal records for *F. hepatica*-damaged liver were available from dairy and beef cattle that were slaughtered in 18 Irish abattoirs between February 2012 and February 2018. Cattle livers were diagnosed as either “liver exhibits *F. hepatica* damage and live *F. hepatica* observed in the liver” or “liver exhibits *F. hepatica* damage without the identifiable presence of live *F. hepatica*”. Animals were considered to have no *F. hepatica*-damaged liver if none of the above diagnostic elements were detected and were slaughtered in the same abattoir and at the same date than an animal diagnosed with a *F. hepatica*-damaged liver. Twomey et al. [8] described in detail the processes to generate *F. hepatica*-damaged liver phenotypes, and also the procedures and criteria imposed to maximise the likelihood that only animals exposed to the parasite were included in the analysis based on *F. hepatica*-damaged liver information available from their herd-mates. In summary, only animals with no herd movement after 90 days of age were retained. Animals were considered as exposed if they had resided in a herd 100 days prior to the slaughter of a herd-mate with live *F. hepatica* present in the liver. If the slaughtered herd-mate had a *F. hepatica*-damaged liver with no observable live *F. hepatica*, an additional criterion was imposed, i.e. as well as having resided in the herd 100 days prior to the slaughter of the slaughtered herd-mate, animals also had to be born within 100 days of the birth of the slaughtered herd-mate, to be considered as exposed.

For the purposes of our study, liver damage caused by *F. hepatica* was dichotomized; animals were considered either infected (i.e., observation of *F. hepatica* damage in the liver) or not infected with *F. hepatica* (i.e., liver did not exhibit *F. hepatica* damage). The final dataset consisted of 187,584 animals with a *F. hepatica*-damaged liver record, either positive or negative.

Breeding values and associated reliabilities for the binary trait of *F. hepatica*-damaged liver were estimated with an animal linear mixed model in the MIX99 genetic evaluation software suite [16]. The model and variance components specified in the genetic evaluations were those previously documented for the genetic evaluation of *F. hepatica*-damaged liver [8]. Fixed effects in the model were parity/age group, stage of lactation, heterosis and recombination coefficients, animal gender, herd-level contemporary group, and date-by-abattoir of slaughter. The random effects were the direct additive genetic effects, where $a\sim\,N\left( {0,{\mathbf{A}}\sigma_{a}^{2} } \right)$ with $\sigma_{a}^{2}$ representing the additive variance and ${\mathbf{A}}$ representing the numerator relationships matrix, and a residual effect, where $e\sim\,N\left( {0,{\mathbf{I}}\sigma_{e}^{2} } \right)$ with $\sigma_{e}^{2}$ representing the residual variance and ${\mathbf{I}}$ representing an identity matrix. The pedigree of each animal was traced back to the founder population, which was allocated to 11 genetic groups based on breed (i.e., Holstein, Friesian, Belgian Blue, Hereford, Aberdeen Angus, Simmental Limousin, Charolais, several other French breeds not listed, and other breeds not listed and unknown); the pedigree consisted of 865,173 animals. The genetic and the residual variances were equal to 0.001 and 0.115, respectively, as estimated by Twomey et al. [8] in a large subset of animals used in the present study. Only EBV for animals that had both a genotype and an EBV reliability higher than 0.01 were considered for further analyses. Moreover, 16,196 animals were retained if they had a record themselves for *F. hepatica*-damaged liver (n = 10,837) or were sires that had at least one progeny record for *F. hepatica*-damaged liver (n = 5359). The effective record contribution (ERC) for each animal was estimated and used as the weighting factor for the deregression of EBV. Deregression was undertaken using the Secant method in the MiX99 software [17]. Deregressed EBV for animals (i.e., a combination of animals with a record themselves and sires with at least one progeny record) with an ERC lower than 1 were not considered further; finally, 3702 animals remained for the analyses.

### Phenotypic data for antibody response to endo-parasites

Blood samples were collected in autumn 2016 from 10,879 dairy cows as part of a cross-sectional seroprevalence study of 68 Irish dairy herds. All blood samples were tested separately for the presence of antibodies to *F. hepatica* using the Svanovir *F. hepatica*-Ab ELISA kit, to *O. ostertagi* using the Svanovir *O. ostertagi*-Ab ELISA kit, and to *N. caninum* using the Svanovir Neospora-Ab ELISA kit (Boehringer Ingelheim Svanova, Uppsala, Sweden). ELISA tests for all blood samples were carried out by the same commercial laboratory (FarmLab Diagnostics, Co. Roscommon, Ireland). Antibody responses to *F. hepatica* and *O. ostertagi* were expressed as optical density ratio (ODR), and antibody response to *N. caninum* was expressed as percent positivity (PP). Data edits were as described by Twomey et al. [10]. In summary, the positively skewed ODR for *F. hepatica* was transformed using the natural logarithm to approximate a normally distributed variable, whereas a reciprocal transformation was used to normalize the positively skewed PP for *N. caninum*. Records were removed if, at blood sampling, the cow resided in a herd different to that in which it had been present at 90 days of age. Only animals with a record for antibody response to *F. hepatica* were retained if they resided in a herd that had at least five cows positive for antibody response to *F. hepatica* (i.e., an ODR ≥ 0.4 was previously shown to lead to production losses [18] and, thus, this cut-off was regarded as the positive threshold for antibody response to *F. hepatica* in the current study) and a within-herd prevalence higher than 5% with a positive antibody response to *F. hepatica* on the day of the blood test. Since the majority of grazing cattle are exposed to *O. ostertagi* [19, 20] and since cows in our study were known to graze grass for most of the year, all cows were considered as exposed to *O. ostertagi*. For antibody response to *N. caninum,* we retained only the records as positive for cows that resided in a herd with a within-herd cow prevalence of more than 1% (i.e., ELISA test manufacturer stated that a PP ≥ 20 indicates a positive result for *N. caninum,* and was thus treated as such in our study) for antibody response to *N. caninum*. In addition, the likelihood that progeny from a cow infected with *N. caninum* will also be infected with *N. caninum* [19] is high, thus, we discarded progeny from dams that had a positive result for *N. caninum* (86 cows were discarded). The final dataset consisted of 6892 records for antibody response to *F. hepatica*, 9260 records for antibody response to *O. ostertagi* and 5289 records for antibody response to *N. caninum*.

Each observed animal phenotype for the continuous traits for antibody response to *F. hepatica*, *O. ostertagi* and *N. caninum* was adjusted for non-genetic effects. Genetic and non-genetic effects for antibody response to each of the three parasites were estimated using the MIX99 software suite [16]. The statistical model was as previously described by Twomey et al. [10]. Fixed effects for antibody response to each of the three parasites were parity, age relative to parity median, stage of lactation, heterosis and recombination coefficients, and contemporary group; random effects were the direct additive genetic effects, where $a\sim\,N\left( {0,{\mathbf{A}}\sigma_{a}^{2} } \right)$ with $\sigma_{a}^{2}$ representing the additive variance and ${\mathbf{A}}$ representing the numerator relationships matrix, and a residual effect, where $e\sim\,N\left( {0,{\mathbf{I}}\sigma_{e}^{2} } \right)$ with $\sigma_{e}^{2}$ representing the residual variance and ${\mathbf{I}}$ representing an identity matrix. The pedigree of each animal was traced back to the founder population, which was allocated to 11 genetic groups based on breed (i.e., Holstein, Friesian, Belgian Blue, Hereford, Aberdeen Angus, Simmental Limousin, Charolais, several other French breeds not listed, and other breeds not listed and unknown). Variance components were as previously described by Twomey et al. [10] for the current dataset; the fitted genetic variance was 0.095 for antibody response to *F. hepatica*, 0.003 for antibody response to *O. ostertagi* and 0.001 for antibody response to *N. caninum*, while the residual variance was 0.734 for *F. hepatica*, 0.034 for *O. ostertagi* and 0.010 for *N. caninum*, which resulted in heritability values of 0.12, 0.08 and 0.09, respectively. For each cow, phenotypes for the three parasites were adjusted for fixed effects (estimated with the linear mixed model) from the cow’s respective phenotype. Only animals that had both an adjusted phenotype and an available genotype were retained for further analysis; 6388 animals remained for *F. hepatica*, 8334 animals remained for *O. ostertagi* and 4597 animals remained for *N. caninum*.

### Genotypes

Genotypes were available from six different genotyping panels, and the number of animals genotyped per panel is in Additional file 1: Table S1. In the dataset of deregressed EBV for *F. hepatica*-damaged liver, 87% of the animals were genotyped using either the Illumina bovine High-Density BeadChip (HD; n = 777,962 SNPs; 20% of animals) or the International Dairy and Beef version 1 (IDBv1; n = 17,137 SNPs; 10% of animals), version 2 (IDBv2; n = 18,004 SNPs; 32% of animals) or version 3 (IDBv3; n = 53,450 SNPs; 25% of animals) genotyping panels. In the dataset of adjusted phenotypes for antibody response to the three different phenotypes, most of the animals were genotyped on the IDBv1 (2% of animals), IDBv2 (76% of animals) or IDBv3 (14% of animals) genotyping panel. All animals had a call rate higher than 90% and only autosomal SNPs, SNPs with a reported chromosomal position, and SNPs with a call rate higher than 90% were retained for each panel.

All animals were imputed to HD using a two-step approach with the FImpute2 software [21]; first, the IDB genotyped animals were imputed to the Bovine SNP50 density (50 k) and subsequently all resulting genotypes (including the Bovine SNP50 genotypes) were imputed to HD using a multi-breed reference population of 5504 HD genotyped animals. Second, imputation to whole-genome sequence (WGS) was undertaken using a multi-breed reference population of 2333 *Bos taurus* animals from Run6.0 of the 1000 Bulls Genomes Project. All variants in the reference population were called using SAMtools, and genotype calls were improved using the Beagle software to provide a consensus SNP density across all animals. Details of the alignment to the UMD 3.1 *Bos taurus* assembly, variant calling and quality controls that were completed within the multi-breed sequence reference population are as described by Daetwyler et al. [22]. In total, 41.39 million SNPs in Run6.0 were identified across the genome and the average coverage (standard deviation) was 12.85 (6.94)X. Imputation of the HD genotypes to WGS was then completed by first phasing all HD genotypes using Eagle ([23]; version 2.3.2) and subsequently imputing to WGS using minimac3 [24].

Regions with poor WGS imputation accuracy, perhaps due to local mis-assemblies or mis-orientated contigs, were identified by using a larger dataset of 147,309 verified parent-progeny relationships. Mendelian errors, defined as the proportion of opposing homozygotes in a parent-progeny pair, were estimated for each relationship and the subsequent Mendelian error rate per SNP was calculated. To accurately identify genomic regions with poor imputation, the R package GenWin [25] which fits a β-spline to the data to find likely inflection points, was used to determine the breakpoints of genomic regions with high Mendelian errors. Windows were analysed using an initial window size of 5 kb and Genwin pooled windows for which the SNP Mendelian error rates were similar. The average SNP Mendelian error rate per window was estimated and all variants within windows in which the mean SNP Mendelian error rate was higher than 0.02 were removed (i.e. 687,137 SNPs were removed).

Further quality control edits were also imposed on the imputed sequence genotypes, including the removal of SNPs with a minor allele frequency (MAF) lower than 0.002 and of SNPs that deviated from Hardy–Weinberg equation (P < 6 × 10^−6^). After these edits, 14,190,141 SNPs remained for animals with a deregressed EBV for *F. hepatica*-damaged liver, whereas 16,603,644 SNPs remained for cows with an antibody response to a parasitic disease.

**Fig. 1:**
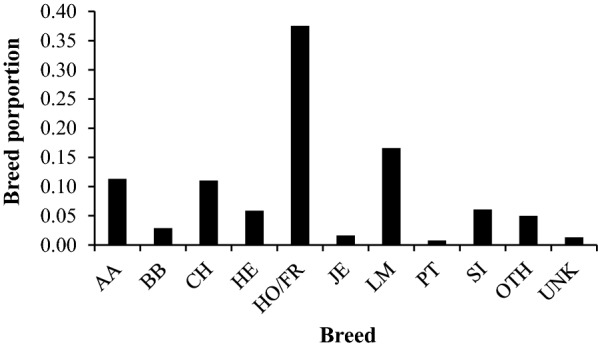
The mean breed composition of animals (n = 3702) in the dataset for *F. hepatica*-damaged liver for Aberdeen Angus (AA), Belgian Blue (BB), Charolais (CH), Hereford (HE), Holstein–Friesian (HO/FR), Jersey (JE), Limousin (LM), Parthenaise (PT), Simmental (SI), all other known breeds (OTH) and unknown breed (UNK)

The final dataset of animals with both a deregressed EBV for *F. hepatica*-damaged and an imputed sequence genotype contained 3702 animals. The mean breed composition of the animals in the dataset of *F. hepatica*-damaged liver was 38% Holstein–Friesian, 17% Limousin, 11% Charolais, 11% Aberdeen Angus and the remaining 23% was another breed (Fig. 1). The final dataset of animals with both an adjusted phenotype for antibody response and an imputed sequence genotype contained 6388 animals for *F. hepatica*, 8334 animals for *O. ostertagi* and 4597 animals for *N. caninum*. In the dataset of antibody response to the three parasitic diseases, the mean breed composition of the cows was 84% Holstein–Friesian, 9% Jersey with the remaining 7% from another breed.

### Association analyses

A genomic relationship matrix was calculated separately for each of the traits using Method I of VanRaden [26] as:

$$\frac{{{\mathbf{ZZ}}^{{\prime }} }}{2\sum pq},$$

where ${\mathbf{Z}}$ is an incidence matrix for the HD genotypes, centred by allele frequencies, and $p$ and $q$ are the dominant and recessive allele, respectively.

For all the traits in this study, SNP effects were estimated using linear mixed models in the WOMBAT software [27]. For each of the dependent variables, animal was included as a random effect with relationships between animals accounted for via the genomic relationship matrix. When the dependent variable was a deregressed EBV for *F. hepatica*-damaged liver, the phenotype was weighted based on the information available. The weight was calculated as [28]:

$$w_{i} = \frac{{1 - h^{2} }}{{\left[ {c + \frac{{1 - r_{i}^{2} }}{{r_{i}^{2} }}} \right]h^{2} }},$$

where $w_{i}$ is the weighting factor of the *i*th deregressed EBV, $h^{2}$ is the heritability estimate for *F. hepatica*-damaged liver, $r_{i}^{2}$ is the reliability of the *i*th deregressed EBV, and $c$ is the proportion of genetic variance not accounted for by the SNPs and was set at 0.9. Purfield et al. [29] tested various values of $c$ (i.e., 0.1, 0.2, 0.8 and 0.9) and reported that all had a minimal impact on the results. The proportions of Holstein–Friesian, Limousin, Charolais, Simmental, Aberdeen Angus, Belgian Blue, Parthenaise, Jersey and other breeds were fitted as covariates in the model for *F. hepatica*-damaged liver; a constraint was imposed by setting the effect of the Hereford breed to zero to avoid linear dependencies. The genomic inflation was estimated for each association analysis and all $\lambda$ estimates were less than 1.1, i.e. 1.004 for *F. hepatica*-damaged liver, 1.0002 for antibody response to *F. hepatica*, 1.0133 for antibody response to *O. osteragi* and 1.0320 for antibody response to *N. caninum*. Multiple-testing correction was applied to each analysis by transforming the p values into their corresponding q values assuming a false discovery rate of 5% [30]. Therefore, SNPs with a q value lower than 0.05 were regarded as significant for the purpose of this study. Suggestive SNPs were defined as SNPs with a p value lower than 10^−5^.

### Quantitative trait loci (QTL) regions

Using PLINK [31], QTL regions were defined around each SNP of interest, based on the flanking LD pattern. For each significant or suggestive SNP, its squared correlation ($r^{2}$) with all the other SNPs within 5 Mb upstream and 5 Mb downstream was calculated. Using a threshold of 0.5 for $r^{2}$, the start of the QTL region was defined as the position of the SNP that was furthest upstream from the significant or suggestive SNP with an $r^{2}$ higher than 0.5; the end of the QTL region was defined as the position of the SNP that was furthest downstream with an $r^{2}$ with the suggestive or significant SNP higher than 0.5. For QTL regions that overlapped, QTL regions were combined into a single QTL region and maximum and minimum positions were set as start and end boundaries.

### Annotation and gene set enrichment analysis

Gene search using Ensembl (http://ensembl.org) on the UMD 3.1 genome build was carried out by focusing on the defined QTL regions. In addition, protein-coding genes, which were identified either as overlapping with or located within QTL regions, were subject to enrichment analysis on The Database for Annotation, Visualization and Integrated Discovery (DAVID) v. 6.8. Functional annotations (Gene Ontology (GO) Biological Process, GO Cellular Component, GO Molecular Function and Kyoto Encyclopedia of Genes and Genomes (KEGG) and Reactome Pathways) were assigned to genes using the functional annotation tool. P values were calculated by EASE (an adoption of the Fisher Exact test to measure the gene-enrichment in annotation terms) to identify functional annotations that were associated with traits of interest.

## Results

**Fig. 2:**
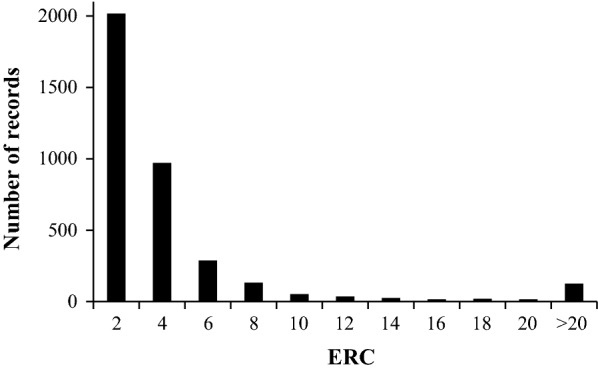
Distribution of effective record contributions (ERC) of animals in the dataset of deregressed *F. hepatica*-damaged liver EBV

The mean effective record contribution (ERC) was equal to 4.53 (ranging from 1.01 to 277.69) in the dataset of *F. hepatica*-damaged liver (Fig. 2).

### *F. hepatica*-damaged liver

**Fig. 3:**
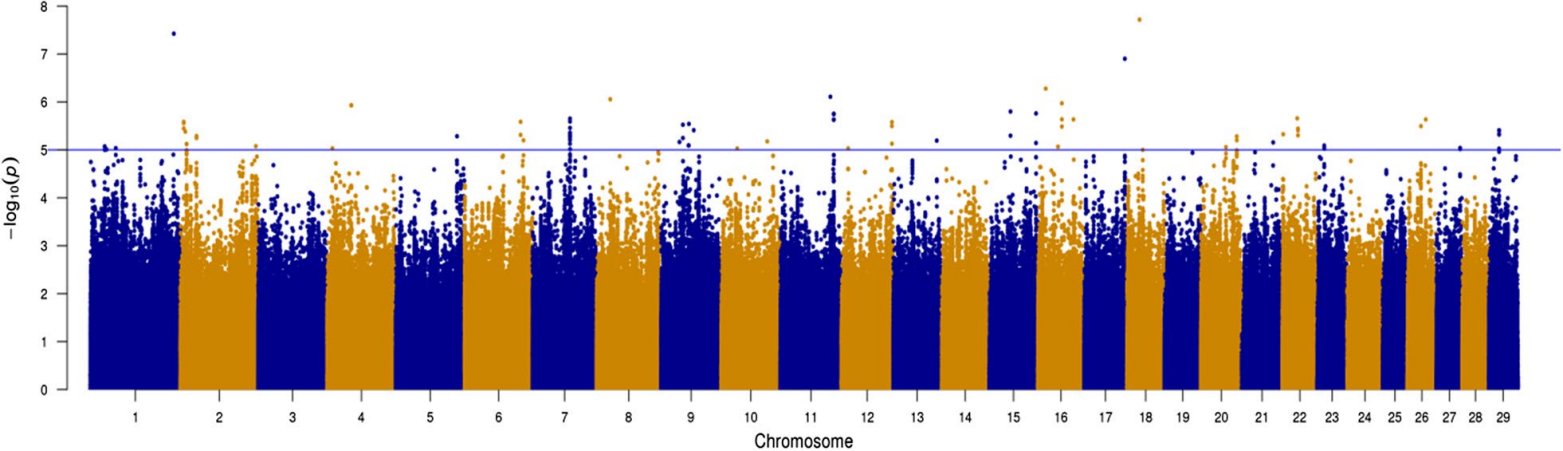
Manhattan plot showing − log_10_(p values) of the association between the effect of each single nucleotide polymorphism (n = 16,603,644 SNPs) and the deregressed estimated breeding value for *F. hepatica*-damaged liver. The blue line is the threshold for suggestive SNPs

The associations between each SNP and *F. hepatica*-damaged liver are shown on the Manhattan plot in Fig. 3. We found no SNP with a q value lower than 0.05 for *F. hepatica*-damaged liver. Details of the most strongly associated SNPs (with a p value < 10^−6^) in each QTL region for *F. hepatica*-damaged liver are in Table 1. Six SNPs, located on BTA1, 8, 11, 16, 17 and 18, had a p value lower than 10^−6^ (Table 1); five of these six SNPs were classified as intronic variants in one of each of the *FTO, AGPAT3, ENSBTAG00000017404, YWHAQ*, or *MLLT3* genes. The sixth SNP for *F. hepatica*-damaged liver was classified as an intergenic variant on BTA1. SNP rs384464701 on BTA18 was the most strongly associated with *F. hepatica*-damaged liver (p value = 1.92 × 10^−8^) and was classified as an intronic variant located in the *FTO* gene. The frequency of the favourable allele for each of the six SNPs with a p value lower than 10^−6^ ranged from 0.002 to 0.998 (Table 1). In total, 121 suggestive SNPs were identified (p value < 10^−5^) and were located across 23 chromosomes.

**Table 1:** Details of the most strongly associated SNPs (with a p value < 10^−6^) in each QTL region with deregressed EBV for *F. hepatica*-damaged liver

BTA	SNP name	Pos (bp)	p value	Gene	Favourable allele (freq)	Effect^a^	Annotation	Start of QTL	End of QTL	Other genes in the QTL region
18	rs384464701	22,279,521	1.92 × 10^−8^	*FTO*	A (0.988)	0.09	Intron	22,279,521	22,279,521	
1	–	146,726,679	3.76 × 10^−8^	*AGPAT3*	G (0.998)	0.18	Intron	146,465,269	14,6811,109	*PDXK*, *CSTB*, *RRP1*, *TRAPPC10*
17	rs110361447	71,554,867	1.26 × 10^−7^	*SEC14L2*	A (0.470)	0.02	Intron	71,554,867	71,554,867	
16	rs800135980	14,007,063	5.27 × 10^−7^	–	A (0.002)	0.19	Intergenic	14,007,063	14,007,063	
11	rs384697649	87,844,503	7.79 × 10^−7^	*YWHAQ*	T (0.996)	0.13	Intron	87,844,503	87,844,503	
8	rs109859381	24,008,154	8.79 × 10^−7^	*MLLT3*	T (0.888)	0.02	Intron	24,008,154	24,008,154	

BTA: *Bos taurus* chromosome number, SNP: name of the single nucleotide polymorphism, pos: position of base pair on the chromosome, p value: p value of the SNP, gene: gene name in which the SNP is located, favourable allele (freq): allele with favourable effect (frequency of the favourable allele), effect: effect of the favourable allele, annotation: annotation of the SNP, start of QTL: base pair on the chromosome at the start of the QTL, end of QTL: base pair on the chromosome at the end of the QTL, other genes in the QTL region: protein-coding genes located in the QTL region that the SNP is not located in

^a^Binary trait (0/1)

Forty-eight QTL regions were suggestively associated with *F. hepatica*-damaged liver (see Additional file 2: Table S2). On BTA7, 21 SNPs were suggestively associated with *F. hepatica*-damaged liver in a single QTL region that spanned from 65.23 to 67.30 Mb and this region included three protein-coding genes (*NMUR2, GRIA1* and *FAM114A2*); the SNP with the strongest association in this region was rs475893299 (p value = 2.23 × 10^−6^). In the QTL region between 93.61 and 93.79 Mb on BTA11, 18 SNPs were suggestively associated with *F. hepatica*-damaged liver; all the genes located in this region encoded olfactory receptors (*OR1J2, OR1N1, ENSBTAG00000047728, ENSBTAG00000047112, ENSBTAG00000046536, ENSBTAG00000045527, ENSBTAG00000045545, ENSBTAG00000020660, ENSBTAG00000038551, OR1Q1,* and *OR1B1*); in addition, the only QTL region associated with antibody response to *F. hepatica* that overlapped with a QTL associated with *F. hepatica*-damaged liver was on BTA11 between 93.74 and 93.89 Mb.

### Antibody response to endo-parasites

**Fig. 4:**
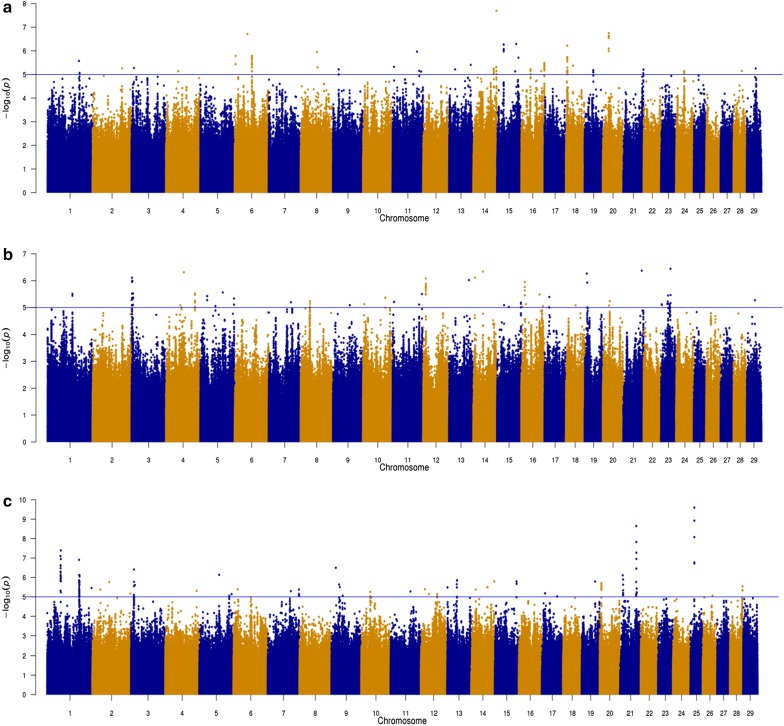
Manhattan plot showing − log_10_(p values) of the association between the effect of each single nucleotide polymorphism and antibody response to **a** *F. hepatica*, **b** *O. ostertagi*, **c** *N. caninum*. The blue line is the threshold for suggestive SNPs

The associations between each SNP and antibody response to *F. hepatica*, *O. ostertagi,* and *N. caninum* are shown on the Manhattan plots in Fig. 4. Details of the most strongly associated SNPs (with a p value < 10^−6^) in each QTL region for antibody response to *F. hepatica*, *O. ostertagi,* and *N. caninum* are in Tables 2, 3 and 4, respectively.

**Table 2:** Details of the most strongly associated SNPs  (with a p value < 10^−6^) in each QTL region with antibody response to *F. hepatica*

BTA	SNP name	Pos (bp)	p value	Gene	Favourable allele (freq)	Effect^a^	Annotation	Start of QTL	End of QTL	Other genes in the QTL region
14	rs801151638	82,704,197	2.02 × 10^−8^	–	A (0.988)	0.42	Intergenic	80,104,349	83,212,505	*RALYL*, *SNX16*, *CHMP4C*
20	rs382942594	21,005,570	1.77 × 10^−7^	–	T (0.063)	0.19	Intergenic	20,574,362	22,008,166	*RAB3C*, *ENSBTAG00000005229, GAPT*, *PLK2*, *ENSBTAG00000026505*
6	rs439064316	44,643,846	1.96 × 10^−7^	–	C (0.008)	0.46	Intergenic	44,643,846	44,643,846	–
15	rs134727358	67,661,877	5.12 × 10^−7^	*PRR5L*	G (0.003)	0.66	Upstream	67,661,877	67,661,877	–
15	rs379765497	23,526,098	5.44 × 10^−7^	–	A (0.008)	0.44	Intergenic	23,515,275	2,3581,425	–
18	rs110443247	4,974,911	6.04 × 10^−7^	–	C (0.375)	0.08	Intergenic	4,974,911	5,158,840	*NUDT7*, *VAT1L*

BTA: *Bos taurus* chromosome number, SNP: name of the single nucleotide polymorphism, pos: position of base pair on the chromosome, p value: p value of the SNP, gene: gene name in which the SNP is located, favourable allele (freq): allele with favourable effect (frequency of the favourable allele), effect: effect of the favourable allele, annotation: annotation of the SNP, start of QTL: base pair on the chromosome at the start of the QTL, end of QTL: base pair on the chromosome at the end of the QTL, other genes in the QTL region: protein-coding genes located in the QTL region that the SNP is not located in

^a^Binary trait (0/1)

**Table 3:** Details of the most strongly associated SNPs (with a p value < 10^−6^) in each QTL region with antibody response to *O. osteratgi*

BTA	SNP name	Pos (bp)	p value	Gene	Favourable allele (freq)	Effect^a^	Annotation	Start of QTL	End of QTL	Other genes in the QTL region
23	rs455561091	33,302,807	3.61 × 10^−7^	–	A (0.004)	0.13	Intergenic	33,029,305	34,481,525	*GPLD1, MRS2, DCDC2, NRSN1, PRP4, PRP8, PRP6*
21	rs475710014	65,072,681	4.26 × 10^−7^	–	A (0.054)	0.03	Intergenic	65,072,681	65,072,681	–
14	rs523857350	35,375,134	4.62 × 10^−7^	–	A (0.005)	0.11	Intergenic	35,375,134	35,740,556	*SULF1, ENSBTAG00000037399, SLCO5A1*
4	rs29026148	62,981,078	4.85 × 10^−7^	–	A (0.650)	0.02	Intergenic	62,981,078	62,993,200	–
19	–	7,584,712	5.43 × 10^−7^	–	A (0.198)	0.02	Intergenic	7,584,712	7,584,712	–
14	rs439873982	7,893,299	7.67 × 10^−7^	–	T (0.008)	0.08	Intergenic	7,866,148	7,899,389	–
3	–	1,746,600	7.74 × 10^−7^	*GPA33*	T (0.004)	0.12	Intron	945,572	6,388,604	*MPZL1, RCSD1, CREG1, CD247, POU2F1, DUSP27, MAEL, ILDR2, TADA1, POGK, ENSBTAG00000022504, ENSBTAG00000022954, FAM78B, UCK2, TMCO1, ALDH9A1, MGST3, LRRC52, RXRG, LMX1A, PBX1, ENSBTAG00000045987, ENSBTAG00000034449, NUF2, RGS5, RGS4*
12	rs379990763	9,621,581	8.25 × 10^−7^	–	A (0.010)	0.08	Intergenic	9,402,063	9,801,565	–
13	rs209722101	70,057,265	9.50 × 10^−7^	–	G (0.002)	0.15	Intergenic	70,057,265	70,057,265	–

BTA: *Bos taurus* chromosome number, SNP: name of the single nucleotide polymorphism, pos: position of base pair on the chromosome, p value: p value of the SNP, gene: gene name in which the SNP is located, favourable allele (freq): allele with favourable effect (frequency of the favourable allele), effect: effect of the favourable allele, annotation: annotation of the SNP, start of QTL: base pair on the chromosome at the start of the QTL, end of QTL: base pair on the chromosome at the end of the QTL, other genes in the QTL region: protein-coding genes located in the QTL region that the SNP is not located in

^a^Binary trait (0/1)

**Table 4:** Details of the most strongly associated SNPs (with a p value < 10^−6^) in each QTL with antibody response to *N. caninum*

BTA	SNP name	Pos (bp)	p value	Gene	Favourable allele (freq)	Effect^a^	Annotation	Start of QTL	End of QTL	Number of genes in the QTL region
25	rs133449464	13,507,716	2.56 × 10^−10^	*PARN*	T (0.005)	0.09	Intron	13,037,560	14,102,058	9
21	–	55,835,765	2.26 × 10^−9^	*PPIP5K1*	A (0.003)	0.11	Intron	55,069,089	56,273,280	29
1	rs466868917	46,464,860	4.08 × 10^−8^	*NXPE3*	C (0.007)	0.08	Intron	45,131,004	49,146,658	18
1	rs381312356	111,186,754	1.24 × 10^−7^	*VEPH1*	C (0.039)	0.03	Intron	106,928,970	113,261,468	32
9	rs714447117	15,859,873	3.19 × 10^−7^	*ENSBTAG00000021175*	C (0.004)	0.07	Downstream	15,859,873	15,859,873	–
3	–	10,934,190	3.89 × 10^−7^	–	C (0.037)	0.03	Intergenic	9,428,852	15,031,457	126
5	rs208471794	70,717,913	7.34 × 10^−7^	–	G (0.929)	0.02	Intergenic	70,717,913	70,717,913	–
21	rs478339448	9,874,248	7.83 × 10^−7^	–	C (0.006)	0.07	Intergenic	9,874,248	10,045,196	–

BTA: *Bos taurus* chromosome number, SNP: name of the single nucleotide polymorphism, pos: position of base pair on the chromosome, p value: p value of the SNP, gene: gene name in which the SNP is located, favourable allele (freq): allele with favourable effect (frequency of the favourable allele), effect: effect of the favourable allele, annotation: annotation of the SNP, start of QTL: base pair on the chromosome at the start of the QTL, end of QTL: base pair on the chromosome at the end of the QTL, other genes in the QTL region: protein-coding genes located in the QTL region that the SNP is not located in

^a^Binary trait (0/1)

### *F. hepatica*

We did not detect any SNP that was significantly associated (q value < 0.05) with antibody response to *F. hepatica*. Fifty-seven SNPs had a p value lower than 10^−6^ and were located on BTA6 (1 SNP), BTA14 (1 SNP), BTA15 (43 SNPs), BTA18 (1 SNP) and BTA20 (11 SNPs). The most highly associated SNP was rs801151638 (p value = 2.02 × 10^−8^), which was classified as an intergenic variant on BTA14 (Table 2). The frequency of the favourable allele ranged from 0.003 to 0.988 for the 57 SNPs with a p value lower than 10^−6^. In total, 226 SNPs were detected as suggestively associated with antibody response to *F. hepatica* (p value < 10^−5^) and 37 QTL regions were identified for antibody response to *F. hepatica;* the largest QTL was 7,593,750 bp long (between 25.26 and 32.86 Mb) on BTA19; (see Additional file 3: Table S3). Some suggestive SNPs were not in linkage disequilibrium (LD; r^2^ < 0.5) with flanking SNPs, thus nine of the 37 detected QTL regions harboured only one SNP. Fifty SNPs were suggestively associated with antibody response to *F. hepatica* in the QTL region between 80.81 and 81.30 Mb on BTA16, which contained 4268 tested SNPs (average LD between 50 suggestive SNPs was 0.99); SNP rs434571591 had the strongest association with antibody response to *F. hepatica* (p value < 3.28 × 10^−6^). Five protein-coding genes were located between 80.8 and 81.30 Mb on BTA16, namely *NR5A2*, *ENSBTAG00000047428*, *KIF14*, *DDX59*, and *CAMSAP2*. In the QTL region between 23.52 and 23.58 Mb on BTA15, 43 of the 709 tested SNPs were detected as suggestively associated with antibody response to *F. hepatica*; no gene was identified in, or overlapped with, this QTL region.

### *O. ostertagi*

We found no SNP significantly associated with antibody response to *O. ostertagi* (q value < 0.05), but nine SNPs were detected for antibody response to *O. ostertagi* with a p value lower than 10^−6^ (Table 3). Of these nine SNPs, seven had a frequency of the favourable allele lower than 0.1, and one SNP was classified as an intronic variant in the *GPA33* gene and the remaining eight as intergenic variants. Forty-two QTL were identified as suggestively associated with antibody response to *O. ostertagi* (p value < 10^−5^; see Additional file 4: Table S4). In total, 134 SNPs were identified as suggestively associated with antibody response to *O. ostertagi.* The QTL region between 94.02 and 98.01 Mb on BTA12 harboured 21 SNPs suggestively associated with antibody response to *O. ostertagi*; no gene was located in this region.

### *N. caninium*

Five SNPs with a q value lower than 0.05 for antibody response to *N. caninium* were located on BTA21 (2 SNPs) and 25 (3 SNPs), respectively. In the QTL region between 13.04 and 14.10 Mb on BTA25, one of the three SNPs was classified as an intronic variant in the *PARN* gene and the other two as downstream variants on the micro-RNA gene, *bta*-*mir*-*365*-*1*. SNP rs133449464, that was classified as an intronic variant in the *PARN* gene, was the most strongly associated (p value = 2.56 × 10^−10^) with antibody response to *N. caninium* (Table 4). The frequency of the favourable allele at SNP rs133449464 was only 0.005 and none of the cows included in the dataset was homozygous for the favourable allele. The two SNPs on BTA21 with a q value lower than 0.05 were located within the QTL between 55.07 and 56.27 Mb and classified as intronic variants in the genes *PPIP5K1* and *PDIA3*; one of these two SNPs was also classified as a downstream variant in the gene *CATSPER2*.

In total, 248 suggestive and significant SNPs were associated with antibody response to *N caninium*; only five of these 248 SNPs had a MAF higher than 0.1. Forty-one QTL were identified as associated with antibody response to *N. caninium*, of which eight contained only one SNP, since the suggestive SNP was not in LD (r^2^ > 0.5) with any other SNP in the flanking region of 5 Mb; the largest of the 41 QTL regions was 6,332,498 bp long (between 106.93 and 113.26 Mb) on BTA1 (see Additional file 5: Table S5). Almost half of the suggestive SNPs associated with antibody response to *N. caninium* were located in only three QTL regions, between 45.13 and 49.15 Mb on BTA1 (24 SNPs), between 106.93 and 113.26 Mb on BTA1 (66 SNPs) and between 57.83 and 58.95 Mb on BTA14 (30 SNPs).

### Gene set enrichment

Of the 102 protein-coding genes, which either overlapped with, or were within QTL regions of suggestive SNPs for *F. hepatica*-damaged liver, DAVID identified 97 for the gene set enrichment analysis. The most strongly associated pathways and gene ontology (GO) sets with *F. hepatica*-damaged liver are in Additional file 6: Table S6. Three pathways and 11 GO sets were identified with a p value lower than 0.05. The pathway R-BTA-381753 had the strongest association with *F. hepatica*-damaged liver and included 11 genes from the gene set. The GO set with the strongest association (p value = 9.50 × 10^−3^) with *F. hepatica*-damaged liver was the biological process of ion transmembrane transport, which included four genes from the gene set.

Gene sets submitted to DAVID consisted of 324 genes for antibody response to *F. hepatica*, 333 genes for antibody response to *O. ostertagi*, and 429 genes for antibody response to *N. caninum*. In total, DAVID recognised 307, 289, and 373 genes for antibody response to *F. hepatica*, *O. ostertagi* and *N. caninum*, respectively. The top five ranked pathways for antibody response to *F. hepatica*, *O. ostertagi,* and *N. caninum* are in Additional file 7: Table S7 and the five GO sets that were most highly associated with antibody response to *F. hepatica*, *O. ostertagi,* and *N. caninum* are in Additional file 8: Table S8.

Five pathways and 28 GO sets were significantly (p value < 0.05) associated with antibody response to *F. hepatica*. The top ranked pathway and GO set for antibody response to *F. hepatica* was R-BTA-2142712 (which included three genes from the gene set; see Additional file 7: Table S7) and lipoxygenase pathway (included six genes from the gene set; see Additional file 8: Table S8), respectively. For antibody response to *O. ostertagi*, we found 14 pathways and 28 GO sets with a p value lower than 0.05. A strong association was identified between the pathway olfactory transduction (p value = 2 × 10^−15^) and antibody response to *O. ostertagi*; 60 genes were included in this pathway and were also identified in QTL regions associated with antibody response to *O. ostertagi* (see Additional file 7: Table S7). The GO biological process of G-protein coupled receptor signalling pathway was strongly associated with antibody response to *O. ostertagi* (p value = 5.40 × 10^−24^; see Additional file 8: Table S8).

In the gene set enrichment analysis of antibody response to *N. caninum*, 19 pathways and 23 GO sets were identified as significant (p value < 0.05). The alpha-linolenic acid metabolism pathway was associated with antibody response to *N. caninum* with a p value lower than 2.70 × 10^−8^ (see Additional file 7: Table S7). The GO metabolic function, phospholipase A2 activity, had the strongest association with antibody response to *N. caninum* (p value = 4.90 × 10^−7^; see Additional file 8: Table S8).

## Discussion

The globally widespread prevalence of endo-parasites in dairy and beef cattle is of major concern [1], since parasitic infection is associated with reduced overall animal performance and compromised animal health and welfare [32, 33]. Although there are many shortcomings of using anthelmintic treatments, a single annual anthelmintic treatment is still the mainstay control strategy for *F. hepatica* and *O. ostertagi* in cattle [9, 34]. For the prevention of *N. caninum* infection, herd biosecurity is the major control strategy adopted in herds [35]. Since several studies have shown that *F. hepatica*, *O. ostertagi,* and *N. caninum* display genetic diversity, genetic selection for resistant cattle could be a complementary approach to current control strategies [8, 10, 11]. In addition, the existence of large genetic variation for endo-parasites in cattle suggests the presence of variability at the genome level, which governs inter-animal variability in susceptibility to endo-parasites. Information at the genome-level, pertaining to the ability of cattle to resist to parasitic infection, could help develop novel pharmaceuticals and diagnostic tests and implement genome-enabled selection. Consequently, knowledge of the biology that underlies such resistance to parasitic infection could help reduce the prevalence of endo-parasites in cattle, and at the same time reduce the reliance on anthelmintic treatments. Therefore, the objective of our study was to locate genomic regions that contribute to the ability of cattle to resist infection to *F. hepatica, O. ostertagi* and *N. caninum*.

### Benefit of using whole-sequence data

It is important to note that our analyses were done on imputed sequence data, which may contain errors [36], and thus to alleviate this issue, rare variants with a MAF lower than 0.2%, SNPs that deviated from Hardy–Weinberg equilibrium (in a larger population to more conclusively identify genotyping errors and abnormalities), and SNPs in regions with a high Mendelian error rate were removed, as described in the Methods section.

To evaluate the benefit of using (imputed) whole-genome sequence in association analyses in cattle, we carried out an additional analysis in which only 50 k and HD SNPs were used (see Additional file 9: Figure S1). When the associations that we detected were limited to only SNPs on either the 50 k or HD panels, no SNP was identified with a p value lower than 10^−6^ for any of the parasite phenotypes investigated. In addition, only 4 and 2% of the QTL detected for antibody response to *F. hepatica* and *O. ostertagi*, respectively, and no QTL for *F. hepatica*-damaged liver and antibody response to *N. caninum* would have been identified if only 50 k SNPs were used instead of the imputed whole-genome sequence data. Furthermore, if we had used only the HD SNPs instead of the imputed whole-sequence data, only 4% of QTL reported here would have been detected for *F. hepatica*-damaged liver and only 11, 5 and 0% of QTL would have been detected for antibody response to *F. hepatica*, *O. ostertagi,* and *N. caninum*, respectively. Therefore, the majority of the causal SNPs or SNPs in LD with causal SNPs for parasitic diseases in cattle are absent on the 50 k and HD panels, which highlights the benefit of using whole-sequence data to identify more precisely the regions of the genome associated with specific traits. It should also be noted, that our study included multiple breeds, which may not be a representative dataset of other studies.

### Comparison to previous genomic studies on parasitic diseases

A limited number of previous studies have attempted to locate genomic regions associated with endo-parasitic diseases in cattle, but none used antibody response or liver damage phenotypes to identify regions of the bovine genome associated with parasitic diseases. In their analysis of 768 Dutch dairy cows using 153 microsatellite markers, Coppieters et al. [11] documented that FEC was associated with the QTL between 22.96 and 25.23 Mb on BTA19 and concluded that the gene located in this region, *ITGAE*, was involved in the immune response to parasitic diseases. Although, in our study, the QTL between 22.96 and 25.23 Mb on BTA19 did not include any suggestive SNPs, a nearby region, located between 25.26 and 32.86 Mb, contained six suggestive SNPs for antibody response to *F. hepatica* (p values ranging from 6.59 × 10^−6^ to 8.09 × 10^−6^). Thus, one or more QTL regions on BTA19 may be associated with resistance to parasitic diseases.

In a study on 584 Angus cattle, Kim et al. [15] reported that 12 regions on the bovine genome contributed to significant genetic variation in FEC; to our knowledge, this is the only previous study that used dense genome-wide SNP genotypes (31,165 SNPs after edits) to map genomic regions associated with endo-parasitic diseases in cattle. In our study, the QTL between 65.23 and 67.30 Mb on BTA7 contained the most suggestive SNPs (21 SNPs) associated with *F. hepatica*-damaged liver, and this region overlaps with one of the QTL (64.19 to 66.89 Mb) identified by Kim et al. [15]. One of the genes that we detected in this region, i.e. *GRIA1*, was previously reported to be associated with ovulation rate in 639 Japanese Black cattle [37]. However, Cushman et al. [38] reported that *GRIA1* had no influence on ovulation rate in a study of 139 crossbred beef cows. In humans, *GRIA1* has been linked to the neurological disease schizophrenia [39, 40], asparaginase hypersensitivity [41], and migraines [42]. In our study, we found that *GRIA1* was involved in two pathways, which were over-represented for *F. hepatica*-damaged liver and are linked to the neurology of the animal, amyotrophic lateral sclerosis, and neuroactive ligand-receptor interaction. Although ectopic *F. hepatica* infection has been reported to cause neurological diseases in humans [43, 44], there are no clinical reports of neurological diseases in cattle associated with lesions in the central nervous system.

Kim et al. [15] also documented a QTL region, located between 48.67 and 66.21 Mb on BTA6, which was suggestively associated with FEC in 584 Aberdeen Angus cattle. In our study, we detected a QTL region (containing 18 suggestive SNPs) on BTA6 associated with antibody response to *F. hepatica* within the region previously reported by Kim et al. [15]. We found four protein-coding genes (*UBE2* *K*, *N4BP2*, *PDS5A*, and *RHOH*) and one RNA gene (*SnRNA*) that were located within this region on BTA6. The *RHOH* gene likely plays a role in the determination of the antibody response to *F. hepatica*, since it is involved in the activation of mast cells, which are known to increase in numbers in response to *F. hepatica* infection [45]. There was no overlap between the associations identified by Kim et al. [15] with FEC and those identified here for antibody response to either *O. ostertagi* or *N. caninum*.

In a study based on only four sheep that were experimentally infected with *F. hepatica*, Alvarez Rojas et al. [46] identified the lipoxygenase pathway with an over-representation of genes that were both up-regulated and down-regulated in peripheral blood mononuclear cells, 2 weeks after infection. These results are supported by our findings on the association of the lipoxygenase pathway and other pathways linked to arachidonic acid with antibody response to *F. hepatica*. This was expected since arachidonic acid, which is a polyunsaturated omega-6 fatty acid, is documented as suppressing the Th1 immune response in mice [47, 48] that decreases during *F. hepatica* infection [49]. Furthermore, studies comparing cows supplemented or not with a range of polyunsaturated fatty acids showed that those that were supplemented had higher levels of T-helper cells, T-cytotoxic cells, cells that expressed IL-2 receptors, and CD62 adhesion molecules [50], as well as a higher mean lymphocyte proliferation and higher titers of anti-OVA IgG [51].

In our study, we also expected that there would be very little overlap between QTL regions associated to the two phenotypes for *F. hepatica*, since Twomey et al. [8] found only a weak genetic relationship between *F. hepatica*-damaged liver and antibody response to *F. hepatica* (0.37; SE = 0.28).

### Regions strongly associated with *N. caninum*

To date, the significant regions associated with antibody response to *N. caninum* on BTA21 and 25 had not been reported to be associated with endo-parasitic diseases in cattle. The favourable alleles for the most significant SNP on BTA21 and 25 had a frequency of only 0.003 and 0.005, respectively, which suggests that, if this frequency increased, more genetic gain is potentially possible. However, when we analysed exclusively the 3038 purebred Holstein–Friesians in our study, the allele frequency of the most significant SNP on BTA21 and 25 dropped to 0.002 and 0.001, respectively and these SNPs were not associated with antibody response to *N. caninum* (p value > 0.06). Nevertheless, the most significant SNP on BTA25 in the multi-breed analysis was an intronic variant located in the *PARN* gene, which encodes an enzyme (PARN) that has a role in early foetal development [52]. In cows, the gene *PARN* has been linked to embryo loss, since the level of *PARN* mRNA was lower in persistent follicles (‘ageing’ follicles associated with embryo loss) than in growing follicles [53]. *N. caninum* infection in females can result in abortion or embryo loss [35] and in reduced reproductive performance [10], thus the association that we observed between PARN and antibody response to *N. caninum* is supported by the association between *PARN* and embryo loss.

### Identification of possible novel candidate genes

We identified that SNP rs384464701, an intronic variant in the *FTO* gene on BTA18, was associated with *F. hepatica*-damaged liver but the frequency of its favourable allele (0.988) was very high, thus, the potential gains for phenotypic improvement in resistance are very limited; in fact, the allele is fixed in the two British breeds of the 301 purebred Aberdeen Angus and 164 purebred Herefords included in our study. Analysis of the 1248 purebred Holstein–Friesians (i.e., ≥ 87.5% Holstein–Friesian) in our study revealed that this SNP rs384464701 had a p value of 3.91 × 10^−4^ and the frequency of its favourable allele was 0.003. *FTO* is known to have a strong association with human obesity [54]. Bravard et al. [55] suggested that *FTO* has a role in the metabolic regulation of the liver. In cattle, variants of the *FTO* gene have previously been associated with both growth and carcass traits [56, 57] as well as with fat and protein yield in milk [58]; taken together, these results suggest that *FTO* may be involved in energy homeostasis. However, Twomey et al. [10] reported that *F. hepatica*-damage liver had little or no genetic association with milk production and carcass merit in cattle.

Regarding the antibody response to *O. ostertagi,* we detected a SNP at 1,746,600 bp located on BTA3 in the *GPA33* gene that was associated with a p value of 7.74 × 10^−7^; there were 17 other suggestive SNPs identified in this region between 0.95 and 6.39 Mb on BTA3. Although, the frequency of the favourable allele (< 0.004) was low for all these suggestive SNPs, *GPA33* is a likely candidate gene associated with antibody response to *O. ostertagi*. Indeed, in a study on mice, Williams et al. [59] reported that *GPA33* was important for the intestinal barrier function, and thus it may have a role in the defence of gastrointestinal nematodes such as *O. ostertagi*.

### Avoidance of parasitic diseases

Research on animal resistance to disease focuses mainly on the immune response [60, 61]. Nevertheless, the coevolution of animals and parasites has led to the development of animal behavioural strategies, including an avoidance strategy, to minimize parasite infection [62]. One such avoidance strategy is avoiding faecal areas caused by an odour cue, since the majority of parasites will be located in areas near (or within) faecal material [62]. In a study on 16 Aberdeen Angus steers, Smith et al. [63] observed that cattle avoided grazing areas contaminated by faeces, with the exception of rabbit faecal material. In a study on 20 sheep, Hutchings et al. [64] reported that ewes classified as genetically resistant to parasitic diseases displayed a different grazing behaviour compared to ewes classified as genetically susceptible to parasitic diseases; ewes that were considered as genetically resistant did not graze areas contaminated by faeces. As suggested by Twomey et al. [8], avoidance strategies may contribute to the genetic differences observed among cattle for different parasite phenotypes. Although, avoidance techniques by livestock may depend on visual clues, odour clues are most likely also involved.

Our findings suggest that cattle use odour cues as part of an avoidance strategy for the three endo-parasites studied here, since at least one pathway and GO set involved in the olfactory system were significant (p value < 0.05) for each of the parasite phenotypes. In addition, the only region on the bovine genome, that contained QTL that were suggestively associated to *F. hepatica*-damaged liver and antibody response to *F. hepatica*, included a large number of olfactory receptor genes. This suggests that a proportion of the genetic variation observed for parasitic diseases may be linked to the ability of cattle to avoid infective larvae. Nevertheless, since genes in these suggestive regions were used in the gene set enrichment analysis, further research is needed to support the concept that cattle avoid infection through odour cues.

## Conclusions

We identified a large number of QTL regions that were associated with the endo-parasite phenotypes investigated in our study. We observed strong associations between genomic regions on BTA21 and 25 with antibody response to *N. caninum*; the associated favourable alleles had a low frequency in the studied population, which suggests that large genetic gain could be achieved.

## Additional files

**Additional file 1: Table S1.** Number of animals with an Illumina Bovine High-Density BeadChip (HD), Illumina Bovine50 beadchip (50 k) and Low-Density BeadChip (LD) and with an International Dairy and Beef version 1 (V1), version 2 (V2) and version 3 (V3) genotype in the current study for deregressed EBV for *F. hepatica*-damaged liver, as well as for the adjusted phenotype of antibody response to *F. hepatica, O. ostertagi* and *N. caninum*.

**Additional file 2: Table S2.** Chromosome number, start of quantitative trait locus (QTL) region, end of QTL region and number of single nucleotide polymorphisms (SNPs) with a p value < 1 × 10^−5^ for each QTL region identified as suggestively associated with *F. hepatica*-damaged liver.

**Additional file 3: Table S3.** Chromosome number, start of quantitative trait locus (QTL) region, end of QTL region and number of single nucleotide polymorphisms (SNPs) with a p value < 1 × 10^−5^ for each QTL region identified as suggestively associated with antibody response to *F. hepatica*.

**Additional file 4: Table S4.** Chromosome number, start of quantitative trait locus (QTL) region, end of QTL region and number of single nucleotide polymorphisms (SNPs) with a p value < 1 × 10^−5^ for each QTL region identified as suggestively associated with antibody response to *O. ostertagi*.

**Additional file 5: Table S5.** Chromosome number, start of quantitative trait locus (QTL) region, end of QTL region and number of single nucleotide polymorphisms (SNPs) with a p value < 1 × 10^−5^ for each QTL region identified as suggestively associated with antibody response to *N. caninium*.

**Additional file 6: Table S6.** Name, type, p value and genes for the top 5 ranked pathways and gene ontology (GO) sets for *F. hepatica*-damaged liver based on the EASE p value (an adoption of the Fisher Exact test to measure the gene-enrichment in annotation terms).

**Additional file 7: Table S7.** Name, source, p value and number of genes for the top 5 ranked pathways for antibody response to *F. hepatica*, *O. ostertagi* and *N. caninum* based on the EASE p value (an adoption of the Fisher Exact test to measure the gene-enrichment in annotation terms).

**Additional file 8: Table S8.** Name, type, p value and genes for the top 5 ranked gene ontology (GO) sets for antibody response to *F. hepatica*, *O. ostertagi* and *N. caninum* based on the EASE p value (an adoption of the Fisher Exact test to measure the gene-enrichment in annotation terms).

**Additional file 9: Figure S1.** Manhattan plot showing -log_10_(p values) of association between each SNP effect and *F. hepatica*-damaged liver for (a) 50 k data and (b) HD data. The blue line is the threshold for suggestive SNPs.
