# Supplementary material for: Genome-wide association study of endo-parasite phenotypes using imputed whole-genome sequence data in dairy and beef cattle
Source: Genet Sel Evol. 2019 Apr 18;51:15. doi: 10.1186/s12711-019-0457-7 (PMC6471778; doi:10.1186/s12711-019-0457-7)
Supplement: Supplementary file 1 — Additional file 1: Table S1. Number of animals with an Illumina Bovine High-Density BeadChip (HD), Illumina Bovine50 beadchip (50 k) and Low-Density BeadChip (LD) and with an International Dairy and Beef version 1 (V1), version 2 (V2) and version 3 (V3) genotype in the current study for deregressed EBV for F. hepatica-damaged liver, as well as for the adjusted phenotype of antibody response to F. hepatica, O. ostertagi and N. caninum. [file 12711_2019_457_MOESM1_ESM.docx]

|  | Illumina Bovine | | |  | International Dairy and Beef | | |
| --- | --- | --- | --- | --- | --- | --- | --- |
|  | HD | 50K | LD |  | V1 | V2 | V3 |
| Deregressed EBVs |  |  |  |  |  |  |  |
| Animals and sires | 731 | 295 | 152 |  | 376 | 1,201 | 947 |
| Adjusted phenotype |  |  |  |  |  |  |  |
| Antibody response to *F. hepatica* |  | 1 | 473 |  | 79 | 4,714 | 1,121 |
| Antibody response to *O. ostertagi* |  | 1 | 703 |  | 143 | 6,335 | 1,152 |
| Antibody response to *N. caninum* |  | 1 | 461 |  | 84 | 3,535 | 516 |
