# Supplementary material for: Genome-wide association study of endo-parasite phenotypes using imputed whole-genome sequence data in dairy and beef cattle
Source: Genet Sel Evol. 2019 Apr 18;51:15. doi: 10.1186/s12711-019-0457-7 (PMC6471778; doi:10.1186/s12711-019-0457-7)
Supplement: Supplementary file 2 — Additional file 2: Table S2. Chromosome number, start of quantitative trait locus (QTL) region, end of QTL region and number of single nucleotide polymorphisms (SNPs) with a p value < 1 × 10^−5^ for each QTL region identified as suggestively associated with F. hepatica-damaged liver. [file 12711_2019_457_MOESM2_ESM.docx]

| Chromosome | Start of QTL | End of QTL | Number of SNPs |
| --- | --- | --- | --- |
| 1 | 25,239,102 | 25,304,726 | 3 |
| 1 | 28,474,337 | 28,474,337 | 1 |
| 1 | 44,704,618 | 44,758,696 | 1 |
| 1 | 146,465,269 | 146,811,109 | 1 |
| 2 | 5,883,407 | 5,887,809 | 4 |
| 2 | 8,656,408 | 8,920,425 | 1 |
| 2 | 10,915,924 | 10,989,211 | 2 |
| 2 | 28,273,173 | 28,346,077 | 3 |
| 2 | 132,716,489 | 132,725,153 | 1 |
| 4 | 9,046,510 | 9,501,875 | 1 |
| 4 | 42,469,234 | 42,503,838 | 3 |
| 5 | 107,554,009 | 107,886,390 | 1 |
| 6 | 98,465,664 | 98,508,838 | 2 |
| 6 | 103,487,450 | 103,683,501 | 1 |
| 7 | 65,229,950 | 67,295,433 | 21 |
| 8 | 24,008,154 | 24,035,059 | 1 |
| 9 | 30,331,290 | 32,573,347 | 1 |
| 9 | 36,464,720 | 40,152,618 | 2 |
| 9 | 48,541,120 | 48,775,446 | 4 |
| 9 | 48,963,948 | 48,972,990 | 1 |
| 9 | 57,537,444 | 57,610,634 | 1 |
| 10 | 28,344,509 | 28,766,213 | 1 |
| 10 | 80,971,000 | 81,432,635 | 1 |
| 11 | 87,844,503 | 87,844,503 | 1 |
| 11 | 93,608,201 | 93,794,295 | 18 |
| 12 | 11,898,866 | 12,141,226 | 1 |
| 12 | 88,815,736 | 88,837,021 | 3 |
| 13 | 76,602,335 | 76,602,335 | 1 |
| 15 | 37,337,211 | 37,337,415 | 2 |
| 15 | 82,354,471 | 82,373,165 | 2 |
| 16 | 14,007,063 | 14,007,063 | 1 |
| 16 | 35,646,927 | 35,646,927 | 1 |
| 16 | 42,351,608 | 42,501,685 | 3 |
| 16 | 62,870,993 | 62,880,174 | 1 |
| 17 | 71,554,867 | 71,554,867 | 1 |
| 18 | 22,279,521 | 22,279,521 | 1 |
| 18 | 27,951,503 | 27,969,992 | 1 |
| 20 | 44,167,645 | 44,336,704 | 1 |
| 20 | 63,317,426 | 63,363,017 | 2 |
| 21 | 55,168,412 | 55,327,559 | 1 |
| 22 | 1,540,724 | 1,540,724 | 1 |
| 22 | 26,100,701 | 26,135,180 | 1 |
| 22 | 27,384,374 | 27,438,589 | 5 |
| 23 | 12,249,499 | 12,330,099 | 3 |
| 26 | 24,280,816 | 24,331,723 | 1 |
| 26 | 32,791,532 | 32,791,532 | 1 |
| 27 | 41,490,582 | 41,624,710 | 2 |
| 29 | 18,262,813 | 18,946,875 | 8 |
