# Supplementary material for: Genome-wide association study of endo-parasite phenotypes using imputed whole-genome sequence data in dairy and beef cattle
Source: Genet Sel Evol. 2019 Apr 18;51:15. doi: 10.1186/s12711-019-0457-7 (PMC6471778; doi:10.1186/s12711-019-0457-7)
Supplement: Supplementary file 3 — Additional file 3: Table S3. Chromosome number, start of quantitative trait locus (QTL) region, end of QTL region and number of single nucleotide polymorphisms (SNPs) with a p value < 1 × 10^−5^ for each QTL region identified as suggestively associated with antibody response to F. hepatica. [file 12711_2019_457_MOESM3_ESM.docx]

| Chromosome | Start of QTL | End of QTL | Number of SNPs |
| --- | --- | --- | --- |
| 1 | 110,564,091 | 113,098,446 | 3 |
| 2 | 103,406,165 | 103,420,468 | 1 |
| 3 | 8,166,175 | 8,522,577 | 1 |
| 4 | 42,796,858 | 42,858,452 | 1 |
| 6 | 3,172,989 | 3,172,989 | 1 |
| 6 | 3,194,525 | 3,228,498 | 9 |
| 6 | 44,643,846 | 44,643,846 | 1 |
| 6 | 60,468,651 | 60,849,818 | 18 |
| 8 | 57,631,941 | 57,631,941 | 1 |
| 8 | 58,760,439 | 61,324,034 | 2 |
| 9 | 20,341,915 | 23,675,403 | 9 |
| 11 | 5,210,652 | 5,213,363 | 1 |
| 11 | 85,967,544 | 86,192,213 | 1 |
| 11 | 93,737,583 | 93,892,928 | 1 |
| 13 | 21,301,056 | 21,401,580 | 1 |
| 13 | 76,540,755 | 76,540,755 | 1 |
| 14 | 73,427,782 | 73,443,137 | 7 |
| 14 | 80,104,349 | 83,212,505 | 6 |
| 15 | 23,515,275 | 23,581,425 | 43 |
| 15 | 63,874,238 | 63,874,238 | 1 |
| 15 | 67,661,877 | 67,661,877 | 1 |
| 15 | 75,286,576 | 75,516,353 | 1 |
| 16 | 32,799,000 | 32,803,837 | 2 |
| 16 | 32,814,558 | 32,870,701 | 6 |
| 16 | 70,192,421 | 70,192,421 | 1 |
| 16 | 80,808,184 | 81,302,245 | 50 |
| 18 | 2,688,728 | 4,584,323 | 1 |
| 18 | 4,974,911 | 5,158,840 | 10 |
| 18 | 5,173,793 | 5,598,390 | 10 |
| 18 | 24,870,642 | 25,417,152 | 2 |
| 19 | 25,262,856 | 32,856,606 | 6 |
| 20 | 20,574,362 | 22,008,166 | 12 |
| 21 | 70,253,070 | 70,336,300 | 1 |
| 21 | 71,055,405 | 71,095,801 | 3 |
| 24 | 28,793,895 | 29,184,221 | 8 |
| 28 | 29,257,551 | 29,257,551 | 1 |
| 29 | 31,481,311 | 31,481,311 | 1 |
