# Supplementary material for: Genome-wide association study of endo-parasite phenotypes using imputed whole-genome sequence data in dairy and beef cattle
Source: Genet Sel Evol. 2019 Apr 18;51:15. doi: 10.1186/s12711-019-0457-7 (PMC6471778; doi:10.1186/s12711-019-0457-7)
Supplement: Supplementary file 4 — Additional file 4: Table S4. Chromosome number, start of quantitative trait locus (QTL) region, end of QTL region and number of single nucleotide polymorphisms (SNPs) with a p value < 1 × 10^−5^ for each QTL region identified as suggestively associated with antibody response to O. ostertagi. [file 12711_2019_457_MOESM4_ESM.docx]

| Chromosome | Start of QTL | End of QTL | Number of SNPs |
| --- | --- | --- | --- |
| 1 | 87,259,516 | 87,510,453 | 2 |
| 3 | 945,572 | 6,388,604 | 18 |
| 4 | 47,262,786 | 51,476,945 | 1 |
| 4 | 62,981,078 | 62,993,200 | 1 |
| 4 | 102,118,878 | 102,296,000 | 10 |
| 5 | 24,417,090 | 24,440,664 | 5 |
| 5 | 53,317,746 | 53,375,155 | 1 |
| 5 | 79,389,656 | 79,938,265 | 1 |
| 5 | 118,632,435 | 119,260,185 | 2 |
| 7 | 78,378,982 | 78,378,982 | 1 |
| 8 | 32,379,291 | 32,649,449 | 3 |
| 9 | 59,217,335 | 59,895,094 | 3 |
| 10 | 430,945 | 9,589,197 | 1 |
| 10 | 78,602,650 | 78,651,058 | 2 |
| 11 | 5,148,122 | 5,195,445 | 3 |
| 11 | 93,123,276 | 93,917,789 | 1 |
| 12 | 9,402,063 | 9,801,565 | 21 |
| 13 | 70,057,265 | 70,057,265 | 1 |
| 14 | 7,866,148 | 7,899,389 | 1 |
| 14 | 35,375,134 | 35,740,556 | 1 |
| 15 | 24,847,021 | 24,847,021 | 1 |
| 15 | 41,922,354 | 41,922,354 | 1 |
| 15 | 84,105,465 | 84,267,130 | 3 |
| 16 | 4,292,961 | 4,469,649 | 1 |
| 16 | 12,057,024 | 12,354,286 | 4 |
| 16 | 16,795,508 | 16,795,532 | 1 |
| 16 | 64,461,579 | 64,674,029 | 1 |
| 16 | 76,308,736 | 76,366,474 | 1 |
| 17 | 16,389,603 | 16,648,154 | 15 |
| 17 | 17,177,818 | 17,216,315 | 2 |
| 18 | 34,503,083 | 34,503,083 | 1 |
| 19 | 7,584,712 | 7,584,712 | 1 |
| 19 | 8,698,129 | 11,829,951 | 2 |
| 20 | 21,449,120 | 21,593,649 | 1 |
| 20 | 24,204,065 | 24,658,248 | 1 |
| 21 | 65,072,681 | 65,072,681 | 1 |
| 23 | 1,946,832 | 1,946,832 | 1 |
| 23 | 2,895,644 | 2,895,644 | 1 |
| 23 | 20,947,816 | 25,162,308 | 4 |
| 23 | 29,403,194 | 31,455,734 | 7 |
| 23 | 33,029,305 | 34,481,525 | 3 |
| 29 | 28,443,385 | 28,992,530 | 1 |
