# Supplementary material for: Genome-wide association study of endo-parasite phenotypes using imputed whole-genome sequence data in dairy and beef cattle
Source: Genet Sel Evol. 2019 Apr 18;51:15. doi: 10.1186/s12711-019-0457-7 (PMC6471778; doi:10.1186/s12711-019-0457-7)
Supplement: Supplementary file 5 — Additional file 5: Table S5. Chromosome number, start of quantitative trait locus (QTL) region, end of QTL region and number of single nucleotide polymorphisms (SNPs) with a p value < 1 × 10^−5^ for each QTL region identified as suggestively associated with antibody response to N. caninium. [file 12711_2019_457_MOESM5_ESM.docx]

| Chromosome | Start of QTL | End of QTL | Number of SNPs |
| --- | --- | --- | --- |
| 1 | 45,131,004 | 49,146,658 | 24 |
| 1 | 106,928,970 | 113,261,468 | 66 |
| 1 | 155,451,980 | 155,451,980 | 1 |
| 2 | 28,237,932 | 28,745,032 | 2 |
| 2 | 59,963,924 | 59,963,924 | 1 |
| 2 | 131,139,965 | 134,492,919 | 1 |
| 3 | 9,428,852 | 15,031,457 | 8 |
| 4 | 111,181,469 | 111,510,760 | 1 |
| 5 | 70,717,913 | 70,717,913 | 1 |
| 5 | 106,491,764 | 106,622,012 | 2 |
| 5 | 114,937,708 | 115,828,907 | 1 |
| 6 | 14,293,982 | 16,018,245 | 1 |
| 7 | 82,530,698 | 82,530,698 | 1 |
| 7 | 111,139,188 | 111,264,101 | 20 |
| 9 | 15,859,873 | 15,859,873 | 1 |
| 9 | 27,455,807 | 27,455,807 | 1 |
| 9 | 27,914,327 | 30,371,007 | 1 |
| 9 | 39,533,254 | 40,898,250 | 1 |
| 10 | 28,579,339 | 33,079,157 | 3 |
| 11 | 70,576,632 | 70,576,656 | 1 |
| 12 | 14,554,295 | 14,615,735 | 1 |
| 12 | 28,973,723 | 28,973,738 | 1 |
| 12 | 55,993,688 | 56,103,585 | 2 |
| 13 | 1,258,717 | 3,319,347 | 2 |
| 13 | 29,660,935 | 34,549,487 | 4 |
| 13 | 35,165,560 | 35,252,189 | 2 |
| 14 | 16,511,565 | 19,616,369 | 1 |
| 14 | 57,831,018 | 58,952,317 | 30 |
| 14 | 83,108,698 | 83,109,951 | 2 |
| 15 | 77,299,567 | 77,545,644 | 3 |
| 17 | 11,379,975 | 12,910,280 | 3 |
| 17 | 54,489,170 | 54,489,170 | 1 |
| 19 | 47,931,754 | 47,931,754 | 1 |
| 20 | 5,914,305 | 6,783,451 | 10 |
| 21 | 9,874,248 | 10,045,196 | 1 |
| 21 | 10,940,476 | 11,314,750 | 15 |
| 21 | 55,069,089 | 56,273,280 | 10 |
| 21 | 57,105,530 | 58,196,401 | 11 |
| 25 | 13,037,560 | 14,102,058 | 5 |
| 26 | 35,338,085 | 35,338,095 | 1 |
| 28 | 44,285,925 | 44,313,933 | 4 |
