# Supplementary material for: Genome-wide association study of endo-parasite phenotypes using imputed whole-genome sequence data in dairy and beef cattle
Source: Genet Sel Evol. 2019 Apr 18;51:15. doi: 10.1186/s12711-019-0457-7 (PMC6471778; doi:10.1186/s12711-019-0457-7)
Supplement: Supplementary file 6 — Additional file 6: Table S6. Name, type, p value and genes for the top 5 ranked pathways and gene ontology (GO) sets for F. hepatica-damaged liver based on the EASE p value (an adoption of the Fisher Exact test to measure the gene-enrichment in annotation terms). [file 12711_2019_457_MOESM6_ESM.docx]

|  | Name | Type | P-value | Genes |
| --- | --- | --- | --- | --- |
| Pathway |  |  |  |  |
|  | R-BTA-381753 |  | 9.50 x 10^-3^ | *OR1N1, ENSBTAG00000045527, OR1B1, ENSBTAG00000045545, OR1J2, OR1Q1, ENSBTAG00000046536, ENSBTAG00000047112* |
|  | R-BTA-888593 |  | 2.40 x 10^-2^ | *SLC6A12, SLC6A13* |
|  | Amyotrophic lateral sclerosis (ALS) |  | 2.80 x 10^-2^ | *GRIA1, TNFRSF1B, RAB5A* |
|  | Neuroactive ligand-receptor interaction |  | 5.30 x 10^-2^ | *CALCRL, CHRM5, GRIA1, GRIK2, NMUR2* |
|  | R-BTA-389359 |  | 7.00 x 10^-2^ | *FYN, PAK1* |
| GO set |  |  |  |  |
|  | Ion transmembrane transport | Biological process | 7.30 x 10^-4^ | *CALHM1, SLC16A10, CALHM2, CALHM3* |
|  | Cation channel activity | Molecular function | 2.20 x 10^-3^ | *CALHM1, CALHM2, CALHM3* |
|  | Plasma membrane | Cellular Component | 5.40 x 10^-3^ | *CYP51A1, MARCKS, KRIT1, NEURL1, CALCRL, PAK1, NMUR2, PTPN13, AQP11, CLNS1A, AGPAT3, ENSBTAG00000020660, OR1N1, ENSBTAG00000038551, ENSBTAG00000045527, OR1B1, ENSBTAG00000045545, OR1J2, OR1Q1,RAB5A, ENSBTAG00000046536, ENSBTAG00000047112* |
|  | G-protein coupled receptor signaling pathway | Biological process | 8.30 x 10^-3^ | *CALCRL, ENSBTAG00000020660, OR1N1, ENSBTAG00000038551, ENSBTAG00000045527, OR1B1, ENSBTAG00000045545, OR1J2, OR1Q1, ENSBTAG00000046536, ENSBTAG00000047112* |
|  | Gamma-aminobutyric acid:sodium symporter activity | Molecular function | 2.20 x 10^-2^ | *SLC6A12 , SLC6A13* |
