# Supplementary material for: Genome-wide association study of endo-parasite phenotypes using imputed whole-genome sequence data in dairy and beef cattle
Source: Genet Sel Evol. 2019 Apr 18;51:15. doi: 10.1186/s12711-019-0457-7 (PMC6471778; doi:10.1186/s12711-019-0457-7)
Supplement: Supplementary file 7 — Additional file 7: Table S7. Name, source, p value and number of genes for the top 5 ranked pathways for antibody response to F. hepatica, O. ostertagi and N. caninum based on the EASE p value (an adoption of the Fisher Exact test to measure the gene-enrichment in annotation terms). [file 12711_2019_457_MOESM7_ESM.docx]

|  | Pathway name | Source | P-value | Genes in pathway |
| --- | --- | --- | --- | --- |
| *F. hepatica* |  |  |  |  |
|  | R-BTA-2142712 | REACTOME | 1.70 x 10^-3^ | *ALOX12B, ALOX12, ALOX15* |
|  | Tight junction | KEGG | 2.40 x 10^-3^ | *MYH2, MYH8, MYH3, F11R, MYH1, CLDN7, MYH10, ENSBTAG00000025337, MYH4* |
|  | Huntington's disease | KEGG | 2.10 x 10^-3^ | *NDUFS2, POLR2A, CREB3, CLTA, DLG4, DNAH2, DNAH9, SDHC, TP53* |
|  | R-BTA-5099900 | REACTOME | 2.70 x 10^-2^ | *CLTA, DVL2, ARRB2* |
|  | Arachidonic acid metabolism | KEGG | 3.60 x 10^-2^ | *ALOX15B, ALOX15, ALOX12B, ALOX12, ALOX12E* |
| *O. ostertagi* |  |  |  |  |
|  | Olfactory transduction | KEGG | 2.00 x 10^-15^ | *ENSBTAG00000000214, OR1J1, OR1L1, ENSBTAG00000004667, ENSBTAG00000005885, ENSBTAG00000013384, ENSBTAG00000015175, ENSBTAG00000020660, ENSBTAG00000027547, ENSBTAG00000027955, ENSBTAG00000031881, ENSBTAG00000035001, ENSBTAG00000037424, ENSBTAG00000037542, ENSBTAG00000037577, ENSBTAG00000037597, ENSBTAG00000037822, ENSBTAG00000038309, ENSBTAG00000038444, ENSBTAG00000038551, ENSBTAG00000038562, ENSBTAG00000038597, ENSBTAG00000038665, ENSBTAG00000038726, ENSBTAG00000038796, ENSBTAG00000038908, ENSBTAG00000038928, ENSBTAG00000038941, ENSBTAG00000039052, ENSBTAG00000039079, ENSBTAG00000039274, OR2B6, ENSBTAG00000040064, ENSBTAG00000040171, ENSBTAG00000040280, ENSBTAG00000040454, ENSBTAG00000040457, ENSBTAG00000040582, ENSBTAG00000040583, ENSBTAG00000045527, OR1B1, ENSBTAG00000045545, ENSBTAG00000045606, ENSBTAG00000045769, ENSBTAG00000045921, ENSBTAG00000046018, OR1J2, ENSBTAG00000046137, OR1Q1, ENSBTAG00000046536, OR10V1, ENSBTAG00000047558, ENSBTAG00000047610, ENSBTAG00000047689, ENSBTAG00000047693, ENSBTAG00000047728, ENSBTAG00000047736, ENSBTAG00000047770, ENSBTAG00000047870, OR1L3* |
|  | R-BTA-381753 | REACTOME | 4.70 x 10^-12^ | *OR1J1, ENSBTAG00000005885, ENSBTAG00000015175, ENSBTAG00000027955, ENSBTAG00000035001, ENSBTAG00000037424, OR1N1, ENSBTAG00000038726, ENSBTAG00000038928, ENSBTAG00000039274, OR2B6,ENSBTAG00000040187, ENSBTAG00000040280, ENSBTAG00000040457, ENSBTAG00000045527, OR1B1, ENSBTAG00000045545, ENSBTAG00000045606, ENSBTAG00000045769, ENSBTAG00000046018, OR1J2, ENSBTAG00000046137, OR1Q1,ENSBTAG00000046536, ENSBTAG00000047112, OR10V1,ENSBTAG00000047689, ENSBTAG00000047693, ENSBTAG00000047770, ENSBTAG00000047870, OR1L3* |
|  | Systemic lupus erythematosus | KEGG | 1.50 x 10^-6^ | *IL10, ENSBTAG00000024175, HIST1H2AG, ENSBTAG00000024177, ENSBTAG00000024178, ENSBTAG00000024180, ENSBTAG00000024182, ENSBTAG00000024183, ENSBTAG00000024186, ENSBTAG00000024187, ENSBTAG00000024188, HIST1H2BB, ENSBTAG00000031888, ENSBTAG00000031889, HIST1H2BN, ENSBTAG00000038277* |
|  | R-BTA-3214847 | REACTOME | 5.50 x 10^-6^ | *ENSBTAG00000031889, ENSBTAG00000024182, ENSBTAG00000024177, ENSBTAG00000024175, ENSBTAG00000024178, ENSBTAG00000024188, ENSBTAG00000038277, HIST1H2BN, ENSBTAG00000024186* |
|  | Alcoholism | KEGG | 1.50 x 10^-5^ | *ENSBTAG00000024175, HIST1H2AG, ENSBTAG00000024177, ENSBTAG00000024178, ENSBTAG00000024180, ENSBTAG00000024182, ENSBTAG00000024183, ENSBTAG00000024186, ENSBTAG00000024187, ENSBTAG00000024188, HIST1H2BB, ENSBTAG00000031888, ENSBTAG00000031889, HIST1H2BN, ENSBTAG00000038277, DRD1* |
| *N. caninium* |  |  |  |  |
|  | alpha-Linolenic acid metabolism | KEGG | 2.70 x 10^-8^ | *PLA2G2A, PLA2G2E, PLA2G2A, PLA2G2F, ENSBTAG00000013039, PLA2G2D1, PLA2G10, PLA2G5, PLA2G2D4* |
|  | Pancreatic secretion | KEGG | 3.30 x 10^-8^ | *PLA2G2A, PLA2G2E, PLA2G2A, PLCB1, ATP1A2, PLA2G2F, ENSBTAG00000013039, CELA3B, PLCB4, PLA2G2D1, PLA2G10, ATP1A4, PLA2G5, PLA2G2D4* |
|  | R-BTA-1482922 | REACTOME | 7.50 x 10^-8^ | *PLA2G2A, PLA2G2E, PLA2G2A, PLA2G2F, ENSBTAG00000013039, PLA2G2D1, PLA2G5, PLA2G2D4* |
|  | R-BTA-1482925 | REACTOME | 1.10 x 10^-7^ | *PLA2G2A, PLA2G2E, PLA2G2A, PLA2G2F, ENSBTAG00000013039, PLA2G2D2, PLA2G6, PLA2G2D5* |
|  | R-BTA-1482801 | REACTOME | 1.70 x 10^-7^ | *PLA2G2A, PLA2G2E, PLA2G2A, PLA2G2F, ENSBTAG00000013039, PLA2G2D3, PLA2G7, PLA2G2D6* |
