# Supplementary material for: Genome-wide association study of endo-parasite phenotypes using imputed whole-genome sequence data in dairy and beef cattle
Source: Genet Sel Evol. 2019 Apr 18;51:15. doi: 10.1186/s12711-019-0457-7 (PMC6471778; doi:10.1186/s12711-019-0457-7)
Supplement: Supplementary file 8 — Additional file 8: Table S8. Name, type, p value and genes for the top 5 ranked gene ontology (GO) sets for antibody response to F. hepatica, O. ostertagi and N. caninum based on the EASE p value (an adoption of the Fisher Exact test to measure the gene-enrichment in annotation terms). [file 12711_2019_457_MOESM8_ESM.docx]

|  | GO set name | Type | P-value | Number of genes in GO set |
| --- | --- | --- | --- | --- |
| *F. hepatica* |  |  |  |  |
|  | Lipoxygenase pathway | Biological Process | 3.00 x 10^-8^ | *ALOX12B, ALOX12, ALOX15, ALOX15B, ALOXE3, ALOX12E* |
|  | Arachidonic acid metabolic process | Biological Process | 1.70 x 10^-6^ | *ALOX12B, ALOX12, ALOX15, ALOX15B, ALOXE3, ALOX12E* |
|  | Linoleic acid metabolic process | Biological Process | 9.80 x 10^-5^ | *ALOX12B, ALOX12, ALOX15, ALOX15B, ALOXE3* |
|  | Arachidonate 12-lipoxygenase activity | Molecular Function | 8.00 x 10^-4^ | *ALOX12B, ALOX12, ALOX15* |
|  | Oxidoreductase activity, acting on single donors with incorporation of molecular oxygen, incorporation of two atoms of oxygen | Molecular Function | 8.70 x 10^-4^ | *ALOX15B, ALOXE3, ALOX12E, P4HA1* |
| *O. ostertagi* |  |  |  |  |
|  | G-protein coupled receptor signaling pathway | Biological Process | 5.40 X 10^-24^ | *ENSBTAG00000000214, OR1J1, OR1L1, ENSBTAG00000004667, ENSBTAG00000005885, ENSBTAG00000013384, ENSBTAG00000015175, ENSBTAG00000020660, ENSBTAG00000027547, ENSBTAG00000027955, ENSBTAG00000031881, ENSBTAG00000035001, ENSBTAG00000037424, ENSBTAG00000037542, ENSBTAG00000037577, ENSBTAG00000037597, ENSBTAG00000037822, ENSBTAG00000038309, ENSBTAG00000038444, ENSBTAG00000038551, ENSBTAG00000038562, ENSBTAG00000038597, ENSBTAG00000038665, ENSBTAG00000038726, ENSBTAG00000038796, ENSBTAG00000038908, ENSBTAG00000038928, ENSBTAG00000038941, ENSBTAG00000039052, ENSBTAG00000039079, ENSBTAG00000039274, OR2B6, ENSBTAG00000040064, ENSBTAG00000040171, ENSBTAG00000040280, ENSBTAG00000040454, ENSBTAG00000040457, ENSBTAG00000040582, ENSBTAG00000040583, ENSBTAG00000045527, OR1B1, ENSBTAG00000045545, ENSBTAG00000045606, ENSBTAG00000045769, ENSBTAG00000045921, ENSBTAG00000046018, OR1J2, ENSBTAG00000046137, OR1Q1, ENSBTAG00000046536, OR10V1, ENSBTAG00000047558, ENSBTAG00000047610, ENSBTAG00000047689, ENSBTAG00000047693, ENSBTAG00000047728, ENSBTAG00000047736, ENSBTAG00000047770, ENSBTAG00000047870, OR1L3* |
|  | G-protein coupled receptor activity | Molecular Function | 9.30 X 10^-23^ | *ENSBTAG00000000214, OR1J1, F2RL2, ENSBTAG00000004667, ENSBTAG00000005885, ENSBTAG00000013384, ENSBTAG00000014795, ENSBTAG00000015175, SFRP4, F2R, ENSBTAG00000020660, ENSBTAG00000027547, ENSBTAG00000027955, ENSBTAG00000031881, F2RL1, ENSBTAG00000035001, ENSBTAG00000037424, ENSBTAG00000037542, ENSBTAG00000037577, ENSBTAG00000037597, OR1N1, ENSBTAG00000038278, ENSBTAG00000038309, ENSBTAG00000038444, ENSBTAG00000038551, ENSBTAG00000038562, ENSBTAG00000038597, ENSBTAG00000038726, ENSBTAG00000038908, ENSBTAG00000038928, ENSBTAG00000038983, ENSBTAG00000039079, ENSBTAG00000039225, ENSBTAG00000039274, OR2B6, ENSBTAG00000040171, ENSBTAG00000040187, ENSBTAG00000040280, ENSBTAG00000040454, ENSBTAG00000040457, ENSBTAG00000040582, ENSBTAG00000040583, ENSBTAG00000045527, OR1B1, ENSBTAG00000045545, ENSBTAG00000045606, ENSBTAG00000045769, ENSBTAG00000046018, OR1J2, ENSBTAG00000046137, OR1Q1, ENSBTAG00000046536, GPR22, ENSBTAG00000047112, OR10V1, ENSBTAG00000047558, ENSBTAG00000047585, ENSBTAG00000047610, ENSBTAG00000047689, ENSBTAG00000047693, ENSBTAG00000047736, ENSBTAG00000047770, ENSBTAG00000047870, OR1L3* |
|  | Olfactory receptor activity | Molecular Function | 3.90 X 10^-22^ | *ENSBTAG00000000214, OR1J1, ENSBTAG00000004667, ENSBTAG00000005885, ENSBTAG00000013384, ENSBTAG00000015175, ENSBTAG00000020660, ENSBTAG00000027547, ENSBTAG00000027955, ENSBTAG00000031881, ENSBTAG00000035001, ENSBTAG00000037424, ENSBTAG00000037542, ENSBTAG00000037577, ENSBTAG00000037597, OR1N1, ENSBTAG00000038278, ENSBTAG00000038309, ENSBTAG00000038444, ENSBTAG00000038551, ENSBTAG00000038562, ENSBTAG00000038597, ENSBTAG00000038726, ENSBTAG00000038908, ENSBTAG00000038928, ENSBTAG00000038983, ENSBTAG00000039079, ENSBTAG00000039225, ENSBTAG00000039274, OR2B6, ENSBTAG00000040171, ENSBTAG00000040187, ENSBTAG00000040280, ENSBTAG00000040454, ENSBTAG00000040457, ENSBTAG00000040582, ENSBTAG00000040583, ENSBTAG00000045527, OR1B1, ENSBTAG00000045545, ENSBTAG00000045606, ENSBTAG00000045769, ENSBTAG00000046018, OR1J2, ENSBTAG00000046137, OR1Q1, ENSBTAG00000046536, ENSBTAG00000047112, OR10V1, ENSBTAG00000047558, ENSBTAG00000047585, ENSBTAG00000047610, ENSBTAG00000047689, ENSBTAG00000047693, ENSBTAG00000047736, ENSBTAG00000047770, ENSBTAG00000047870, OR1L3* |
|  | Sensory perception of smell | Molecular Function | 4.90 X 10^-16^ | *ENSBTAG00000038562, ENSBTAG00000038444, ENSBTAG00000037577, ENSBTAG00000047736, ENSBTAG00000040582, ENSBTAG00000039274, ENSBTAG00000005885, ENSBTAG00000047558, ENSBTAG00000040171, ENSBTAG00000040454, ENSBTAG00000038928, ENSBTAG00000040187, ENSBTAG00000027547, ENSBTAG00000037597, ENSBTAG00000040457, OR2B6, ENSBTAG00000038983, ENSBTAG00000040280, ENSBTAG00000015175, ENSBTAG00000027955, ENSBTAG00000031881, ENSBTAG00000038908, ENSBTAG00000039079, ENSBTAG00000040583, ENSBTAG00000038597, ENSBTAG00000000214, ENSBTAG00000047585* |
|  | Plasma membrane | Cellular Component | 5.70 X 10^-11^ | *ENSBTAG00000000214, OR1J1, F2RL2, ESAM, TICAM2, ENSBTAG00000004667, ENSBTAG00000005885, CFTR, NRCAM, WNT2, GPA33, PANX3, KCNN2, RHAG, VMP1, CD247, ENSBTAG00000013384, PRKAR2B, ENSBTAG00000015175, RGS5, LVRN, F2R, ENSBTAG00000020660, PIK3CG, CDHR3, PAQR8, ENSBTAG00000027547, ENSBTAG00000027955, ENSBTAG00000031881, F2RL1, ENSBTAG00000035001, MCC, ENSBTAG00000037424, ENSBTAG00000037542, ENSBTAG00000037577, ENSBTAG00000037597, OR1N1, ENSBTAG00000038278, ENSBTAG00000038309, , ENSBTAG00000038444, ENSBTAG00000038551, ENSBTAG00000038562, ENSBTAG00000038597, ENSBTAG00000038726, ENSBTAG00000038908, ENSBTAG00000038928, ENSBTAG00000038983, ENSBTAG00000039079, ENSBTAG00000039225, ENSBTAG00000039274, OR2B6, ENSBTAG00000040171, ENSBTAG00000040187, ENSBTAG00000040280, ENSBTAG00000040454, ENSBTAG00000040457, ENSBTAG00000040582, ENSBTAG00000040583, ENSBTAG00000045527, OR1B1, ENSBTAG00000045545, ENSBTAG00000045606, ENSBTAG00000045769, ENSBTAG00000046018, OR1J2, ENSBTAG00000046137, OR1Q1, ENSBTAG00000046536, ENSBTAG00000047112, OR10V1, ENSBTAG00000047558, ENSBTAG00000047585, ENSBTAG00000047610, ENSBTAG00000047689, ENSBTAG00000047693, ENSBTAG00000047736, ENSBTAG00000047770, ENSBTAG00000047870, OR1L3* |
| *N. caninium* |  |  |  |  |
|  | Phospholipase A2 activity | Metabolic function | 4.90 x 10^-7^ | *PLA2G2A, PLA2G2E, PLA2G2A, PLA2G2F, ENSBTAG00000013039, PLA2G2D1, PLA2G5, PLA2G2D4* |
|  | Arachidonic acid secretion | Biological process | 1.70 x 10^-6^ | *PLA2G2A, PLA2G2E, PLA2G2A, PLA2G2F, ENSBTAG00000013039, PLA2G2D1, PLA2G5, PLA2G2D4* |
|  | Lipid catabolic process | Biological process | 1.80 x 10^-6^ | *PLA2G2A, PLA2G2E, PLA2G2A, PLCH1, PLCB1, PLA2G2F, ENSBTAG00000013039, PLCB4, PLA2G2D1, PLA2G5, PLA2G2D4* |
|  | Phospholipid metabolic process | Biological process | 8.10 x 10^-6^ | *PLA2G2A, PLA2G2E, PLA2G2A, PLA2G2F, ENSBTAG00000013039, PLA2G2D1, PLA2G5, PLA2G2D4* |
|  | Transmembrane signaling receptor activity | Metabolic function | 2.70 x 10^-5^ | *OR6K3, ACKR1, OR10T2, OR10J3, OR6K6, ENSBTAG00000017778, ENSBTAG00000020765, ENSBTAG00000020838, ENSBTAG00000022528, OR10K2, ENSBTAG00000037546, ENSBTAG00000037575, MRC1, OR6K2, ENSBTAG00000048292* |
