# Supplementary figures and images for: Genome-wide association study of endo-parasite phenotypes using imputed whole-genome sequence data in dairy and beef cattle
Source: Genet Sel Evol. 2019 Apr 18;51:15. doi: 10.1186/s12711-019-0457-7 (PMC6471778; doi:10.1186/s12711-019-0457-7)

**
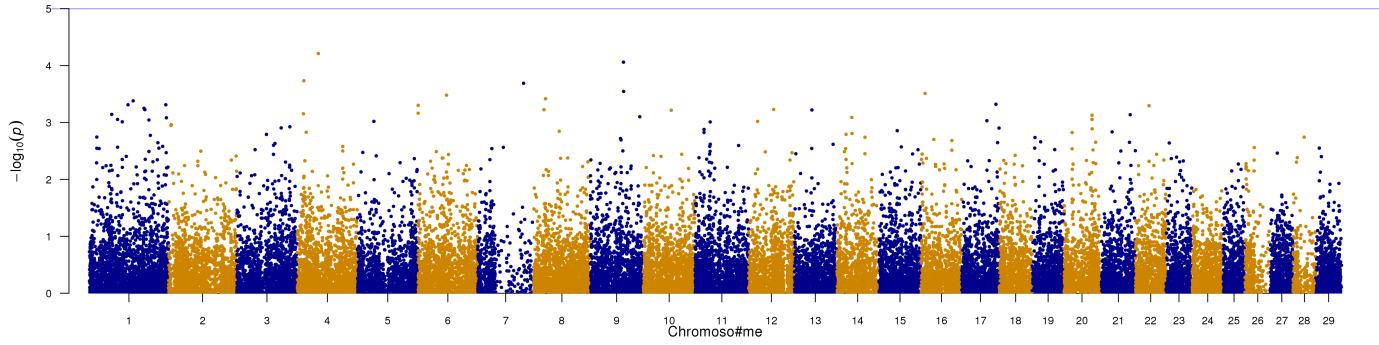
**

**(a)**

**
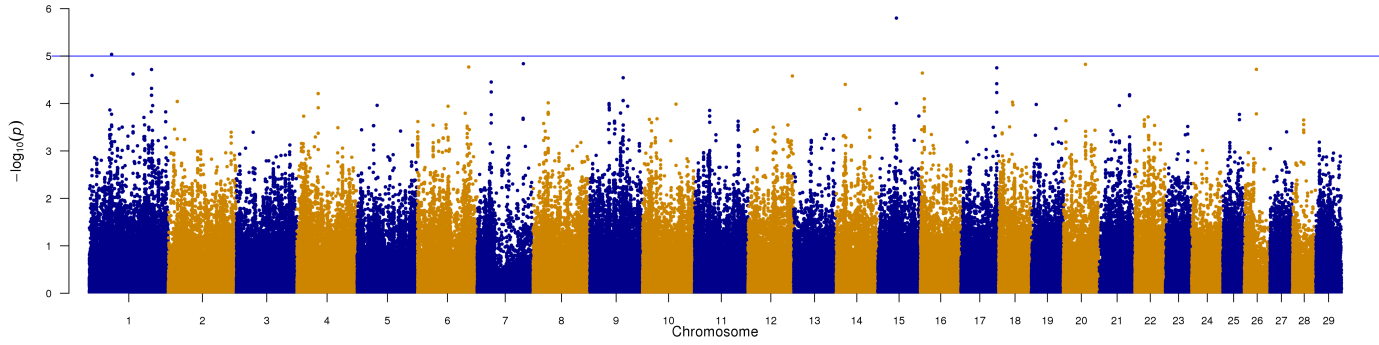
**

**(b)**

Supplement: Supplementary file 9 — Additional file 9: Figure S1. Manhattan plot showing -log10(p values) of association between each SNP effect and F. hepatica-damaged liver for (a) 50 k data and (b) HD data. The blue line is the threshold for suggestive SNPs. [file 12711_2019_457_MOESM9_ESM.docx]
